# What are the main sources of smoking cessation support used by adolescent smokers in England? A cross-sectional study

**DOI:** 10.1186/s12889-015-1925-9

**Published:** 2015-06-19

**Authors:** Wasif Shaikh, Manjula D Nugawela, Lisa Szatkowski

**Affiliations:** School of Medicine, Division of Epidemiology and Public Health, Clinical Sciences Building, Nottingham City Hospital, University of Nottingham, NG5 1PB Nottingham, UK

**Keywords:** Smoking cessation, Adolescents

## Abstract

**Background:**

Adolescent smoking is a worldwide public health concern. Whilst various support measures are available to help young smokers quit, their utilization of cessation support remains unknown.

**Methods:**

A cross-sectional study was conducted using data from the 2012 *Smoking, Drinking and Drug Use among Young People* survey to quantify the use of seven different types of cessation support by adolescents aged 11-16 in England who reported current smoking and having tried to quit, or ex-smoking. Logistic regression was used to calculate odds ratios and 95 % confidence intervals for the associations between participant characteristics and reported use of cessation support.

**Results:**

Amongst 617 current and ex-smokers, 67.3 % (95 % CI 63.0-71.2) reported use of at least one cessation support measure. Not spending time with friends who smoke was the most commonly-used measure, reported by 45.4 % of participants (95 % CI 41.1-49.8), followed by seeking smoking cessation advice from family or friends (27.4 %, 95 % CI 23.7-31.5) and using nicotine products (15.4 %, 95 % CI 12.6-18.7). Support services provided by the National Health Service (NHS) were infrequently utilized. Having received lessons on smoking was significantly associated with reported use of cessation support (adjusted OR 1.55, 95 % CI 1.02-2.34) and not spending time with friends who smoked (adjusted OR 1.98, 95 % CI 1.33-2.95). Students with family members who smoked were more likely to report asking family or friends for help to quit (adjusted OR 1.74, 95 % CI 1.07-2.81). Respondents who smoked fewer cigarettes per week were generally less likely to report use of cessation support measures.

**Conclusion:**

The majority of young smokers reported supported attempts to quit, though the support they used tended to be informal rather than formal. Evidence is needed to quantify the effectiveness of cessation support mechanisms which are acceptable to and used by young smokers.

## Background

Tobacco smoking remains the leading global cause of avoidable mortality [1]. The majority of adult smokers start to smoke during their teenage years [2] and smoking in adolescence is associated with an increased risk of being a heavier smoker in adulthood [3], being less likely to quit [4], and dying of a smoking-related cause [5]. In 2012, 23 % of adolescents aged 11-16 in England reported that they had smoked at least once in their lifetime and 4 % were classified as regular smokers (defined as smoking at least once per week) [6].

A large body of existing literature has examined the effectiveness of smoking cessation interventions in adults, such as the use of nicotine replacement therapy [7], physician advice [8], telephone quitlines [9] and National Health Service (NHS) Stop Smoking Services [10]. However, there remains a paucity of good quality evidence for the effectiveness of cessation support mechanisms in young people [11]. Approximately one third of regular adolescent smokers in England report wanting to give up smoking altogether and nearly two-thirds report having made a cessation attempt [6]. However, little is known about how adolescent smokers who want to quit go about trying to do so, and whether they use any of the sources of cessation support available to them in the process. In conjunction with evidence for effectiveness, an understanding of what means of cessation support are utilised by, and thus likely acceptable to, young people is crucial in planning and targeting cessation services for this age group.

In this study we quantify the self-reported use of different sources of smoking cessation support by adolescents aged 11-16 years in England. We also investigate whether particular individual-level characteristics are associated with adolescents’ reported use of cessation support.

## Methods

### Data source and study population

A cross-sectional study was conducted using data from the 2012 *Smoking, Drinking and Drug Use among Young People* survey. This survey is a school-based repeated cross-sectional study conducted annually to estimate the prevalence of smoking among young people in school Year 7 (aged 11-12) to Year 11 (aged 15-16) in England through confidential self-completion questionnaires. The survey used multi-stage probability sampling to select schools by geographical region (response rate 49 % of selected schools), and students within schools (response rate 88 % of eligible students). Parents were able to opt their child out of completing the survey, and otherwise their consent was assumed. Further details of the survey methods are available elsewhere [6]. The anonymised survey dataset was obtained from the UK Data Service [12]; ethical approval was not required for the use of the data.

From the 2012 survey dataset we identified all respondents who were self-reported regular smokers (currently smoking 1 or more cigarettes per week) who reported ever having tried to quit, as well as ex-regular smokers (who used to smoke 1 or more cigarettes per week). Pupils who were non-smokers or those who reported only having smoked once in their lifetime were excluded.

Data were available for current and ex-smokers’ reported use of seven different methods of cessation support, all categorised as binary (yes/no) responses:

1. asked an adult at school for help to quit;
2. asked family or friends for help to quit;
3. used nicotine products (in the UK nicotine replacement therapies are licensed for prescription by a doctor to children aged 12+ and children over this age are also able to purchase these products over-the-counter without parental consent);
4. visited a General Practitioner (GP);
5. phoned the National Health Service (NHS) smoking helpline (adolescents are able to access the helpline confidentially and anonymously);
6. used NHS Stop Smoking Services (use is confidential and does not require parental consent);
7. not spend time with friends who smoke.

Data were also extracted on a range of individual-level characteristics hypothesised to be potentially associated with adolescents’ use of smoking cessation support: age (school Year 7 to Year 11); sex; weekly cigarette consumption and number of years smoked (both available for current smokers only, the latter split at the median); whether or not they had played truant, or been excluded from school, in last 12 months; whether any of the people the adolescent lived with smoked; whether they had received lessons at school on smoking in last 12 months; and reported cannabis use (the drug most-frequently used by survey respondents) and alcohol consumption. Finally, an indicator of personal well-being was derived from the level of agreement with five statements each measured on a 5-point scale: my life is going well; my life is just right; I wish I had a different kind of life; I have a good life; I have what I want in life. In line with the approach and category labels used by the survey designers, a total score of 0-9 was used to indicate low wellbeing, and 10-20 to indicate not low wellbeing [6].

### Statistical analysis

The percentage of respondents reporting use of each type of cessation support was calculated, for smokers and ex-smokers separately, and then combined. Univariable logistic regression was used to calculate odds ratios and 95 % confidence intervals (CIs) for the associations between student characteristics and reported use of cessation support. Variables which were statistically significant, or nearing statistical significance, based on a p-value of 0.05, were entered into a multivariable model for each type of cessation support. Likelihood Ratio Tests were used to examine the effect of removing variables one at a time in order to derive parsimonious models.

All analyses used survey weights to account for the unequal probability of selection of students to the sample by sex and school year. Data management and analysis was performed using Stata 13 (Stata Corp., College Station, Texas, USA).

## Results

From a total of 7,589 survey respondents, 291 current smokers who reported ever having tried to quit smoking were identified, as well as 326 ex-regular smokers. Table 1 shows the prevalence of use of different smoking cessation support mechanisms reported by the study population.

**Table 1:** Prevalence of use of different types of smoking cessation support

Type of smoking cessation support	Use among current smokers who have tried to quit % (95 % CI)	Use among ex-smokers % (95 % CI)	Use in both groups combined % (95 % CI)
Asked an adult at school for help	11.4 (7.9-16.1)	3.9 (1.8-6.9)	7.2 (5.2-9.8)
Asked family or friends for help	40.9 (34.8-47.4)	15.9 (12.0-20.7)	27.4 (23.7-31.5)
Used nicotine products	24.2 (19.3-29.9)	7.9 (5.3-11.7)	15.4 (12.6-18.7)
Visited a general practitioner (GP)	5.0 (2.9-8.6)	0.8 (0.2-3.1)	2.8 (1.7-4.6)
Phoned the NHS smoking helpline	2.2 (0.9-4.9)	0.4 (0.1-1.6)	1.2 (0.6-2.5)
Used NHS Stop Smoking Services	5.5 (3.3-9.0)	1.3 (0.5-3.6)	3.2 (2.0-5.1)
Not spent time with friends who smoke	42.7 (36.5-49.1)	47.8 (41.9-53.8)	45.4 (41.1-49.8)
Any of the above	76.3 (70.6-81.3)	59.5 (53.5-65.2)	67.3 (63.0-71.2)

Overall, 67.3 % (95 % CI 63.0-71.2) of the study population reported having used one or more of the seven named types of support to quit smoking. The most popular means of support was choosing not to spend time with friends who smoke, reported by 45.4 % of participants. Just over one quarter of the study population reported having asked family or friends for help to quit smoking (27.4 %, 95 % CI 23.7-31.5) and 15.4 % reported that they had used nicotine products (95 % CI 12.6-18.7).

In contrast, relatively few adolescents reported that they had asked an adult at school for help to quit smoking, had visited their GP, or had utilised the cessation support services offered by the NHS. Given the low use of these services, these variables were excluded from subsequent analysis.

Table 2 shows odds ratios for the univariable associations between characteristics of the study participants and their use of support.

**Table 2:** Univariable associations between adolescent characteristics and their use of support

Characteristic	n^*^	Used any means of support		Not spent time with friends		Asked family or friends for help		Used nicotine products	
		OR (95 % CI)	*p*-value	OR (95 % CI)	*p*-value	OR (95 % CI)	*p*-value	OR (95 % CI)	*p*-value
Sex									
Male	304	1.00	0.101	1.00	0.248	1.00	0.179	1.00	0.459
Female	313	1.37 (0.94-1.99)		1.23 (0.87-1.75)		1.31 (0.88-1.95)		1.19 (0.75-1.90)	
School Year									
Year 11	261	1.00	0.256	1.00	0.841	1.00	0.387	1.00	0.847
Year 10	192	0.70 (0.46-1.09)		0.91 (0.60-1.37)		0.71 (0.45-1.12)		0.76 (0.43-1.33)	
Year 9	112	0.64 (0.38-1.08)		0.85 (0.51-1.40)		0.74 (0.41-1.32)		1.07 (0.57-2.01)	
Year 8	43	1.20 (0.55-2.61)		1.06 (0.53-2.11)		0.52 (0.23-1.18)		0.79 (0.31-2.05)	
Year 7	9	0.53 (0.12-2.26)		0.48 (0.11-2.16)		1.09 (0.23-5.13)		0.96 (0.19-4.98)	
Amount smoked (amongst current smokers only)									
Usually >6 cigarettes per week	128	1.00	<0.001	1.00	0.007	1.00	<0.001	1.00	<0.001
Usually 1-6 cigarettes per week	66	0.43 (0.21-0.91)		0.92 (0.46-1.83)		0.46 (0.24-0.89)		0.73 (0.36-1.47)	
<1 cigarette per week	97	0.83 (0.42-1.64)		2.47 (1.35-4.51)		0.44 (0.24-0.81)		0.24 (0.11-0.54)	
Years since first cigarette (amongst current smokers only)									
0-2	181	1.00	0.335	1.00	0.042	1.00	0.906	1.00	0.031
3+	91	0.72 (0.38-1.4)		0.54 (0.30-0.98)		1.03 (0.59-1.83)		1.96 (1.07-3.62)	
Truanted in last 12 months									
No	379	1.00	0.822	1.00	0.374	1.00	0.940	1.00	0.032
Yes	199	0.95 (0.63-1.43)		0.84 (0.57-1.23)		0.98 (0.64-1.51)		1.70 (1.05-2.76)	
Excluded in last 12 months									
No	451	1.00	0.361	1.00	0.048	1.00	0.092	1.00	0.018
Yes	131	1.24 (0.78-1.95)		0.63 (0.40-1.00)		1.52 (0.93-2.46)		1.89 (1.11-3.20)	
Wellbeing									
Not low wellbeing	370	1.00	0.143	1.00	0.336	1.00	0.360	1.00	0.207
Low wellbeing	173	1.39 (0.89-2.17)		1.21 (0.82-1.82)		1.23 (0.79-1.93)		1.39 (0.83-2.31)	
Whether any of the people the child lives with smoke themselves (*e.g.* parent, sibling)									
No	199	1.00	0.932	1.00	0.225	1.00	0.005	1.00	0.027
Yes	370	0.98 (0.65-1.48)		0.79 (0.54-1.16)		1.91 (1.21-3.00)		1.80 (1.07-3.03)	
Had lessons on smoking in the last 12 months									
No	201	1.00	0.038	1.00	0.001	1.00	0.870	1.00	0.900
Yes	338	1.55 (1.02-2.33)		1.98 (1.33-2.94)		0.96 (0.62-1.49)		1.03 (0.61-1.74)	
Cannabis use									
No	294	1.00	0.720	1.00	0.066	1.00	0.142	1.00	0.271
Yes	305	0.93 (0.64-1.36)		0.72 (0.50-1.02)		1.35 (0.90-2.01)		1.30 (0.81-2.10)	
Frequency of drinking alcohol									
At least once a week	165	1.00	0.125	1.00	0.066	1.00	0.892	1.00	0.155
At least once a month	204	0.79 (0.50-1.27)		0.96 (0.61-1.52)		0.92 (0.55-1.52)		0.58 (0.32-1.07)	
Less frequently/never	207	1.27 (0.79-2.06)		1.53 (0.97-2.41)		1.02 (0.62-1.70)		0.62 (0.35-1.14)	

^*^number may not equal total as data for participants with missing data have not been presented for brevity

Figures in bold are statistically significant (*p* < 0.05)

At univariable level there were no statistically significant associations between participant age, sex, wellbeing, cannabis and alcohol use and students’ use of any, or specific types, of smoking cessation support. Participants who had truanted, or been excluded from school, in the last 12 months were approaching twice as likely to report having used nicotine products to help them quit smoking compared to those who had not. Participants who lived with a smoker were again approaching twice as likely to report having asked family or friends for help to quit, or to report having used nicotine products, than those who did not live with a smoker. Participants who had received lessons on smoking in the last 12 months were more likely to report use of one or more means of support than those who had not (OR 1.55, 95 % CI 1.02-2.33) and were almost twice as likely to report not spending time with friends who smoke. Compared to current smokers who usually smoked more than six cigarettes per week, those who smoked less, or who were ex-smokers were less likely to report using any means of support, asking family or friends for support, or using nicotine products. However, those who smoked less than one cigarette per week or who were ex-smokers were more likely to report not spending time with friends who smoked. Current smokers who had been smoking for at least three years were less likely to report not spending time with friends who smoked, but more likely to report having used nicotine products, compared to those who started smoking more recently.

The associations between truanting, school exclusion, duration of smoking and reported use of cessation support were attenuated in the multivariable models, with amount smoked and either living with a smoker or receiving lessons on smoking in the last 12 months being the only variables significantly associated with the outcomes (Table 3).

**Table 3:** Multivariable associations between student characteristics and use of support

Characteristic	Used any means of support		Not spent time with friends		Asked family or friends for help		Used nicotine products	
	OR (95 % CI)	*p*-value	OR (95 % CI)	*p*-value	OR (95 % CI)	*p*-value	OR (95 % CI)	*p*-value
Amount smoked (amongst current smokers only)								
Usually >6 cigarettes per week	1.00	<0.001	1.00	0.006	1.00	<0.001	1.00	<0.001
Usually 1-6 cigarettes per week	0.43 (0.21-0.91)		0.92 (0.46-1.84)		0.50 (0.26-0.98)		0.73 (0.36-1.47)	
<1 cigarette per week	0.83 (0.42-1.65)		2.50 (1.36-4.60)		0.47 (0.25-0.86)		0.24 (0.11-0.54)	
Ex-smokers	0.34 (0.21-0.57)		1.68 (1.04-2.71)		0.18 (0.11-0.30)		0.17 (0.10-0.31)	
Whether any of the people the child lives with smoke themselves (*e.g.* parent, sibling)								
No	n/s		n/s		1.00	0.024	n/s	
Yes					1.74 (1.07-2.81)			
Had lessons on smoking in the last 12 months								
No	1.00	0.041	1.00	0.001	n/s		n/s	
Yes	1.55 (1.02-2.34)		1.98 (1.33-2.95)					

n/s = not significant in multivariable model

## Discussion and conclusions

To the best of our knowledge this is the first study to quantify young smokers’ use of cessation support. Our findings show that whilst approximately two thirds of current and ex-smokers reported having used one or more means of cessation support, the most frequently utilised mechanisms were quite informal, such as seeking advice from friends and family or choosing not to spend time with friends who smoke. More formal avenues of support, such as seeking advice from a GP, or using the NHS telephone or face-to-face smoking cessation services, were very infrequently used. At present there is little evidence that young people’s use of pharmacotherapy can increase quit success [11]; further work is warranted in this area given that a relatively large proportion (15.4 %) of participants in this study, particularly those who smoked more cigarettes per week, reported having used nicotine products to help them quit. Further work would also be of use to understand the effectiveness of approaches to promoting cessation which combine different methods of support; there is some evidence that complex interventions [11], and those engaging with young smokers over a more extended period of time [13], may be effective in achieving cessation.

The major strength of this study was the use of a representative sample of young people aged 11-16 living in England, though caution is warranted when generalizing the results of this study to young people living elsewhere. Unfortunately, the cross-sectional design of the *Smoking, Drinking and Drug Use in Young People* survey, and the nature of the questions asked, means we were not able to investigate whether reported use of particular types of quit support is associated with successful cessation. Cohort studies or randomised controlled trials may help to provide further evidence to support recommendations for the use of particular types of cessation support in this age group. We were also limited in our investigation of factors associated with use of cessation support to variables on which data were available from the *Smoking, Drinking and Drug Use in Young People* survey. No data were available on the extent of addiction of smoking, though this has previously been shown to be associated with cessation [14]. Similarly, other studies have demonstrated an association between frequency of adolescent drug and alcohol use and making a quit attempt [15]; we found no significant associations between reported substance use and use of cessation support, though our definition of substance use was less detailed compared to other studies as we were not able to identify frequency of cannabis use or binge drinking from the survey dataset.

In adults there is evidence to suggest that unaided quit attempts are the least likely to succeed [16]. If future research suggests the same holds true in the age group studied here, then attention should be paid to understanding which young people are least likely to utilise support when trying to quit, as well as considering how to increase uptake of support in these groups. The results presented here show that current smokers who smoked fewer cigarettes per week were less likely to report use of cessation support than heavier smokers. Further work is warranted to investigate attitudes towards smoking cessation in young people who smoke relatively few cigarettes per week and how to ensure successful cessation in this group. Adolescents who had received lessons on smoking in the last 12 months were more likely to report overall use of cessation support and choosing not to spend time with friends who smoke. If this is shown to be an effective way of enabling and supporting successful cessation then this would justify continued promotion of, and investment in, the delivery of smoking education in schools. In England, however, questions have been raised about the quality of Personal, Social, Health and Economic Education (PSHE) [17], of which teaching about smoking is a part.

In conclusion, this study has shown that the majority of young smokers proactively use several means of support when trying to quit. More evidence is needed to quantify the effectiveness of cessation support mechanisms which are acceptable to young smokers.

## Abbreviations

95 % CI: 95 % Confidence interval
NHS: National Health Service
OR: Odds ratio
PSHE: Personal, Social, Health and Economic Education
